# A bibliometric analysis of the advance of artificial intelligence in medicine

**DOI:** 10.3389/fmed.2025.1504428

**Published:** 2025-02-21

**Authors:** Mian Lin, Lingzhi Lin, Lingling Lin, Zhengqiu Lin, Xiaoxiao Yan

**Affiliations:** ^1^Department of Orthopedics, The Third Affiliated Hospital of Wenzhou Medical University, Wenzhou, China; ^2^Department of Neurology, The Third Affiliated Hospital of Wenzhou Medical University, Wenzhou, China

**Keywords:** artificial intelligence, medicine, bibliometrics, VOSviewer, applications

## Abstract

**Introduction:**

The integration of artificial intelligence (AI) into medicine has ushered an era of unprecedented innovation, with substantial impacts on healthcare delivery and patient outcomes. Understanding the current development, primary research focuses, and key contributors in AI applications in medicine through bibliometric analysis is essential.

**Methods:**

For this research, we utilized the Web of Science Core Collection as our main database and performed a review of literature covering the period from January 2019 to December 2023. VOSviewer and R-bibliometrix were performed to conduct bibliometric analysis and network visualization, including the number of publications, countries, journals, citations, authors, and keywords.

**Results:**

A total of 1,811 publications on research for AI in medicine were released across 565 journals by 12,376 authors affiliated with 3,583 institutions from 97 countries. The United States became the foremost producer of scholarly works, significantly impacting the field. Harvard Medical School exhibited the highest publication count among all institutions. The Journal of Medical Internet Research achieved the highest H-index (19), publication count (76), and total citations (1,495). Four keyword clusters were identified, covering AI applications in digital health, COVID-19 and ChatGPT, precision medicine, and public health epidemiology. “Outcomes” and “Risk” demonstrated a notable upward trend, indicating the utilization of AI in engaging with clinicians and patients to discuss patients’ health condition risks, foreshadowing future research focal points.

**Conclusion:**

Analyzing our bibliometric data allowed us to identify progress, focus areas, and emerging fields in AI for medicine, pointing to potential future research directions. Since 2019, there has been a steady rise in publications related to AI in medicine, indicating its rapid growth. In addition, we reviewed journals and significant publications to pinpoint prominent countries, institutions, and academics. Researchers will gain important insights into the current landscape, collaborative frameworks, and key research topics in the field from this study. The findings suggest directions for future research.

## 1 Introduction

Artificial intelligence (AI) involves interpreting information and analyzing the application of algorithms. Advanced computer algorithms are utilized in AI to perform tasks such as decision-making and data interpretation, similar to humans. AI offers diverse options for identifying and solving various problems. Like humans, AI machines have the capacity for critical thinking. AI operates through multiple pathways, enabling systems to detect new patterns and derive different formulations from given data. Ongoing AI development has transformed medical practice significantly, transitioning from traditional methods to digital healthcare. Leveraging its advanced algorithms and deep learning capabilities, AI has become a valuable tool for physicians and healthcare providers, aiding in various aspects such as health information management, geolocation of health data, disease surveillance, predictive analytics, decision-making support, and medical imaging (1–4). As a significant cause of death and disability around the world, stroke presents a considerable threat to public health. Neuroimaging plays a significant role in stroke research, and CT scans are the usual choice for examining patients suspected of having a stroke. In a study involving 477 patients, CT angiography was processed using an automatic detection algorithm, obtaining a diagnostic sensitivity of 94% and a negative predictive value of 98% in merely 5 min (5). So, AI markedly improved diagnostic efficiency, especially for patients who required transfer to comprehensive stroke centers for thrombectomy, thus reducing further brain damage and enhancing their prognosis. Furthermore, The ability of AI to detect infarction regions was enhanced with a 1–4 h interval from symptom onset to imaging, emphasizing its potential for early intervention (6). This development is being applied to the entire cardiovascular medicine sector, which is increasingly using AI technologies. Machine learning (ML) represents a vital component of AI, facilitating autonomous learning from data by algorithms.

Machine learning includes methods like linear regression, logistic regression, support vector machines (SVM), and decision trees (7). ML is applied in numerous sectors, including the analysis of medical images, the prediction of patient prognoses, and the formulation of personalized treatment plans (8). Tasks that are repetitive or manually intensive, like validating general chemistry test results or analyzing blood cells and urine cultures, have seen improved efficiency due to ML. Research involving continuous glucose monitoring (CGM) data for blood glucose prediction has indicated that ML can predict type 1 diabetes with accuracy rates surpassing 90% (9, 10) and inaccuracies in clinical laboratory test outcomes, like blood placed in incorrect tubes, mislabeled samples, and contamination, have been detected using ML during clinical lab testing (11). Thus, in laboratory medicine, ML has been researched to enhance the precision and dependability of test outcomes. Besides, AI systems can offer healthcare professionals continuous, and potentially instantaneous, access to medical updates from a multitude of sources, including academic journals, medical texts, clinical experiences, and patient data (12). This facilitates informed clinical decision-making, enables accurate forecasting of health outcomes, and enables accurate health risk alerts and outcome predictions (13). Medical research shows that AI holds more promise than other fields when it comes to output-input ratio (14).

Bibliometric analysis examines its structure, quantity, and impact. Researchers, institutions, countries, or specific fields of research may be analyzed. It employs mathematical and probabilistic methods to retrieve and study information from academic journals. Bibliometric endeavors to discover trends, patterns, and developments in research literature. A significant impact of this analysis is in relation to the appraisal of academic performance, research productivity, and distribution of resources (15, 16). Lately, numerous global bibliometric studies have been conducted with the help of CiteSpace and VOSviewer. The analyses have concentrated on the overall rehabilitation statuses and research trends concerning diseases such as cancer, ankylosing spondylitis, motor and neuropathic pain, and osteoarthritis (17, 18). As AI becomes a crucial tool in medicine, it is important to comprehend its influence and evolution in the scientific domain. Nonetheless, the significant research and development in this field pose a challenge: the importance of systematically reviewing and measuring the rise of scientific literature on AI in healthcare. So our research investigates article characteristics on AI in medicine over the past 5 years, reflecting a growing interest in this domain. This surge in interest aligns with the heightened awareness of AI’s significance in risk assessment, diagnosis, treatment, and prevention of diseases. Comparing to previous studies, the value of this research is found in its methodical review and integration of existing literature, effectively charting the complex network of research endeavors and collaborative efforts. By delineating the current knowledge landscape, this mapping creates a foundation for subsequent research and acts as a pivotal reference for researchers, medical practitioners, and policymakers, steering their initiatives toward the integration of AI in medicine, construction a knowledge graph in this area to deliver valuable insights for future investigations.

## 2 Materials and methods

### 2.1 Data source and search methodology

**FIGURE 1:**
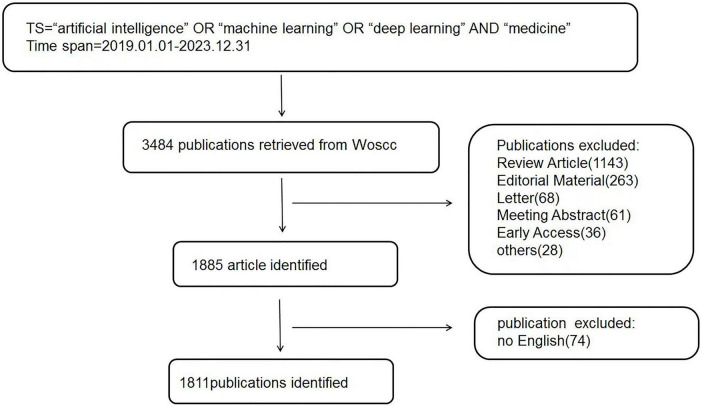
Flowchart depicting the inclusion and exclusion process for literary research.

A comprehensive search methodology was employed to search for works on the subject of AI in medicine from the Web of Science Core Collection (WoSCC) database spanning from January 2019 to December 2023. More than 21,000 journals in science, social sciences, and humanities can be accessed via the WoSCC (19). We chose this database for the multidisciplinary nature and citation tracking, which aids in identifying the most powerfull AI in medicine publications. It was possible to locate publications pertaining to the incidence, causes, genetic aspects, symptoms, identification, and treatment of this disorder by accessing this database. WoSCC stands out as a significant online database and is viewed as the most ideal for bibliometric analysis (19). The search strategy was: TI = (“artificial intelligence” OR “machine learning” OR “deep learning” AND “medicine”). It was retrieved between January 2019 and December 2023. Our search was limited to “article” documents. Only English-language papers were included in the search. The diagram outlining the process of choosing publications are show in Figure 1.

### 2.2 Data retrieval and analysis

Sources and data extracted from WoSCC files, in txt or BibTeX format, were imported into VOSviewer 1.6.18 and R 4.0.2 for analysis and visualization. The extracted data include information on authorship, institutions, publications, keywords, and other relevant details essential for bibliometric analysis. This data extraction methodology ensures the acquisition of current and relevant information in AI applied to medicine, enabling a comprehensive and representative analysis.

## 3 Results

### 3.1 The annual pattern of publication growth

**FIGURE 2:**
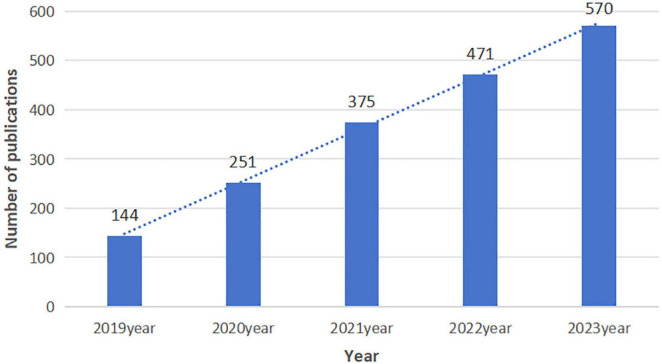
The trend of publications about AI in medicine.

The WoSCC database yielded 1,811 articles centered on AI in the medical field. These reports were collectively authored by 12,376 individuals representing 3,583 organizations across 97 countries. A total of 565 journals published these works, which collectively referenced 73,095 citations from 15,180 journals. Over the previous 5 years, there has been a rise in the number of publications, which has grown by 28.4% each year, indicating the field’s developmental trend. This growth is visually depicted in Figure 2, illustrating a consistent year-on-year increase in publication output.

### 3.2 Geographical distribution

The leading 10 countries in AI for medical research are outlined in Table 1, detailing their publication count (NP), total citations (NC), and average citations (AC). The United States ranked first in both publications and citations, contributing 709 papers (39.09%) and 14,764 citations. China followed with 371 papers (31.41%), and England with 189 papers (16.00%). It is noteworthy that despite China ranking second in NP, but the AC was relatively lower compared to other top 10 productive countries.

**TABLE 1:** Displays the breakdown of the leading 10 countries.

Order	Country	Number of publications	Number of citations	Average citation
1	United States	708	14,764	20.85
2	China	371	3,547	9.56
3	England	189	5,889	31.16
4	Germany	176	3,567	20.27
5	Italy	143	2,142	14.98
6	Canada	124	2,882	23.24
7	South Korea	102	959	9.40
8	Netherlands	98	3,150	32.14
9	France	92	1,573	17.10
10	Spain	77	1,302	16.91

**FIGURE 3:**
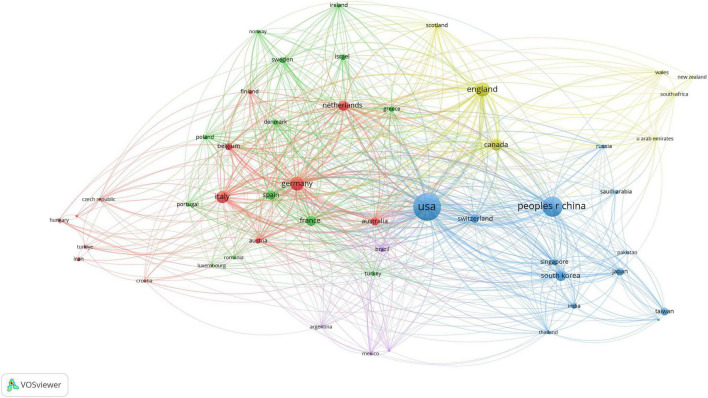
Visualization of countries’ relationships.

The study of scientific cooperation involves scholars working collectively to generate new scientific knowledge. Figure 3 presents the co-authorship analysis, with node size representing the number of articles published by each country. There is a close collaboration between nodes, with a wider line indicating more intensity. With 624 links and a total link strength of 2,653, the co-authorship network analysis of AI in medicine shows an organized structure with five clusters and strong global collaboration. Leading this collaboration are the United States, China, England, Germany, and Italy. Based on Table 2, the foremost five authors and co-cited authors have played a crucial role in contributing to AI research in medicine. The author with the most articles (15) is Li J, and the author with the most citations (20) is Ho MT.

**TABLE 2:** An overview of the top five authors and their co-citations.

Rank	Authors	Publications	Rank	Co-cited authors	Citations
1	Li J	15	1	Ho MT	24
2	Wang J	13	2	Corrado G	23
3	Zhang J	12	3	Karthikesalingam A	23
4	Zhang H	12	4	Kelly CJ	23
5	Liu Y	12	5	King D	23

The wide range of geographical locations represented in the network highlights the strong global cooperation in the industry, which promotes a variety of viewpoints and expertise in research. These results give significant viewpoints on the patterns in AI research in the medical field and emphasize the potential for increased cooperation among specific nations.

#### 3.3 Analysis of journal articles

**FIGURE 4:**
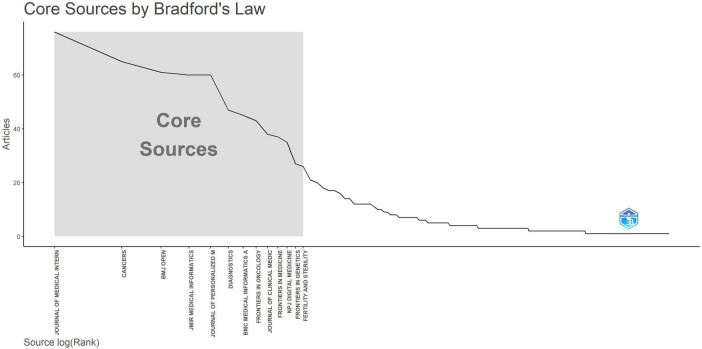
Bradford’s law identifies primary sources.

As presented in Table 3, we collected the NC and publication output from the top 10 sources with the highest H-index in the area of AI in medicine. Notably, the Journal of Medical Internet Research exhibited the highest H-index (H-index = 19) and publication count (NP = 76). Applying Bradford’s law to the 1,495 sources, we identified 13 journals, including Journal of Medical, Cancers, BMJ Open, JMIR Medical Informatics, and Journal of Personalized Medicine, as core journals as a result of t their relatively high publication output (Figure 4).

**TABLE 3:** Leading 10 journals and their co-cited counterparts.

Rank	Journal	H-index	NP	NC	PYS
1	Journal of Medical Internet Research	19	76	1,495	2019
2	NPJ Digital Medicine	17	35	1,807	2019
3	Cancers	15	65	670	2019
4	Fertility and Sterility	14	26	470	2019
5	BMC Medical Informatics and Decision Making	12	45	933	2019
6	JMIR Medical Informatics	12	60	569	2019
7	Journal of Personalized Medicine	12	60	488	2020
8	Diagnostics	11	47	385	2020
9	Lancet Digital Health	11	12	898	2020
10	Frontiers in Oncology	10	43	312	2019

NP, publication count; NC, total citations; PYS, publication year start.

#### 3.4 Analysis of the institution

**FIGURE 5:**
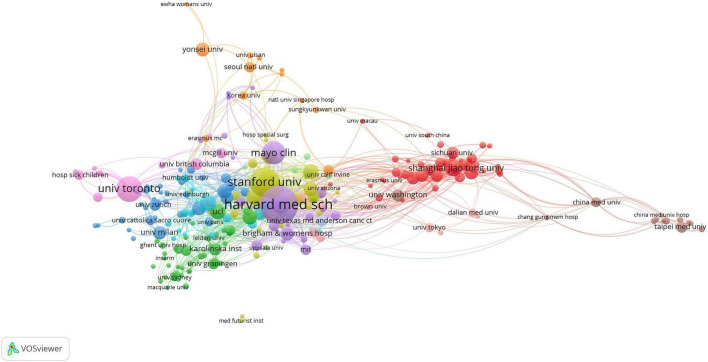
Scientific collaboration between institutions.

To evaluate institutional importance and collaboration, we developed a network plot (Figure 5). Institutions are ranked by the size of their nodes and the level of their activity, and collaboration between them is measured by the thickness of their lines. Research institutions in AI medicine were grouped into 12 primary clusters. Institutions with the greatest prominence and activity were Harvard Medical School, followed by Stanford University, University of Toronto, Mayo Clinic, and Johns Hopkins University. Among the top five institutions, four are located in the United States, with the exception of the University of Toronto in Canada. Harvard Medical School exhibited the highest level of collaboration. Shanghai Jiao Tong University had the highest centrality in China, followed by Sichuan University and South China University. Furthermore, these institutions were central to facilitating collaboration within the same groups. Additionally, Stanford University and Harvard Medical School showed significant disparities in their arrangement and distribution within the co-occurrence network. With a clustering coefficient of 12, research demonstrates unique characteristics, advantages, or distinct directions.

### 3.5 Top referenced research papers

Table 4 shows the 10 papers that are most frequently cited in the field of AI in medicine, representing significant contributions and perhaps the most renowned works in this domain. Normalized Local Citations (NLC) serve to mitigate variations in citation counts due to differences in academic disciplines and publication dates. The article “Key challenges for delivering clinical impact with artificial intelligence,” authored by Kelly et al. (5) from Google Health, London, United Kingdom, and published in BMC Medicine in 2019, holds the highest NLC (16.98). This article emphasizes the exciting opportunity presented by AI to enhance healthcare. It stresses the essential need for robust, prospective clinical evaluation to ensure the safety and effectiveness of AI systems. Such evaluation should employ clinically applicable performance metrics that extend beyond technical accuracy to encompass the impact of AI on care quality, healthcare professionals’ variability, clinical practice efficiency and productivity, and patient outcomes (5). Closely following in the NLC ranking is the article “The state of artificial intelligence-based FDA-approved medical devices and algorithms: an online database,” authored by Benjamens et al. (21) from the Netherlands in 2020. This article examines FDA-approved AI-based medical devices and algorithms.

**TABLE 4:** The 10 most frequently cited local papers on the topic.

Document	DOI	Year	Normalized local citations
Kelly CJ, 2019, BMC Med	10.1186/s12916-019-1426-2	2019	16.98
Sidey-Gibbons JAM, 2019, BMC Med Res Methodol	10.1186/s12874-019-0681-4	2019	9.60
Benjamens S, 2020, NPJ Digit Med	10.1038/s41746-020-00324-0	2020	16.40
Tran BX, 2019, J Clin Med	10.3390/jcm8030360	2019	8.86
Bi WL, 2019, CA-Cancer J Clin	10.3322/caac.21553	2019	7.38
Oh S, 2019, J Med Internet Res	10.2196/12422	2019	7.38
Zaninovic N, 2020, Fertil Steril	10.1016/j.fertnstert.2020.09.157	2020	12.61
Guo YQ, 2020, J Med Internet Res	10.2196/18228	2020	10.09
Ghorbani A, 2020, NPJ Digit Med	10.1038/s41746-019-0216-8	2020	10.09
Cikes M, 2019, Eur J Heart Fail	10.1002/ejhf.1333	2019	5.17

**FIGURE 6:**
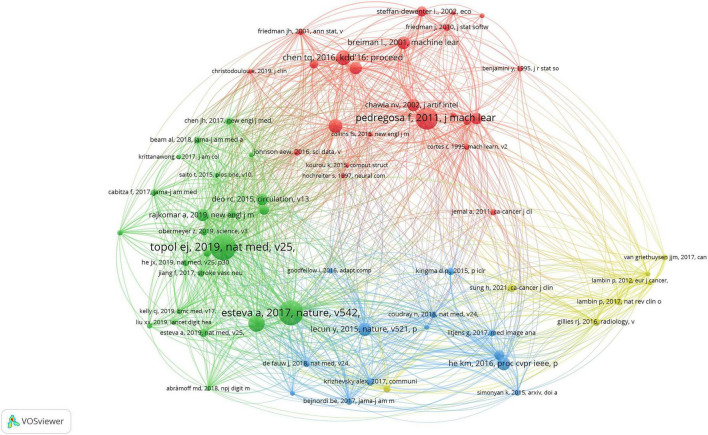
The analysis of co-cited documents.

Document co-citation identifies literature that is frequently cited together by different authors. This method visualizes citation co-occurrence between two publications to assess their connection (22). Figure 6 illustrates a co-citation reference map for AI in medicine. In 2019, the paper that received the most citations, totaling 110, was “High-performance medicine: The convergence of human and artificial intelligence” by Topol (23). This article highlighted AI’s influence in medicine on three fronts: aiding clinicians with swift and precise image analysis; improving health systems by streamlining processes and minimizing medical mistakes; and empowering patients with self-data processing. The second-ranked paper illustrates skin lesion classification utilizing Deep Convolutional Neural Networks (CNNs) (24). With a dataset of 129,450 clinical images and 2,032 diseases, the authors trained a CNN that is much larger than previous datasets. The CNN’s classification of skin cancer matches the performance of dermatologists in both tasks.

### 3.6 Analysis of trend topics and keywords

**FIGURE 7:**
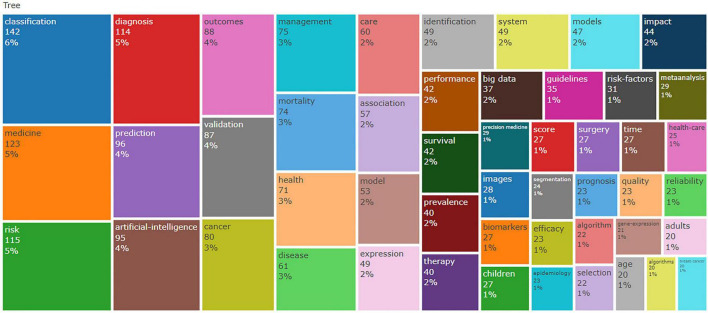
Tree Map of the keyword.

**FIGURE 8:**
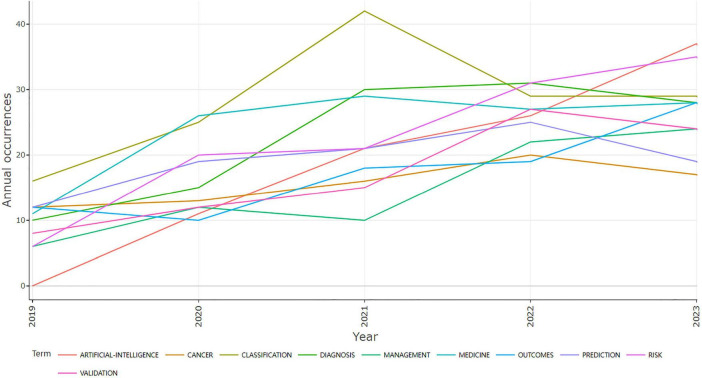
Time series of author keywords.

Figure 7 illustrates the most frequently used keywords, in Figure 8, the use patterns and trends of author keywords are summarized. The keyword “Classification” emerged as the most frequently used term after 2019, but has experienced a slight decline since 2021. Its usage surged significantly, reflecting an increased research focus in this area. Furthermore, keywords such as “Cancer,” “Diagnosis,” and “Prediction” gained high popularity from 2019 to 2022. Since 2022, the keywords “Artificial intelligence,” “Outcomes,” and “Risk” have exhibited a notable increase in usage, indicating their promise as developing focal points in research.

**FIGURE 9:**
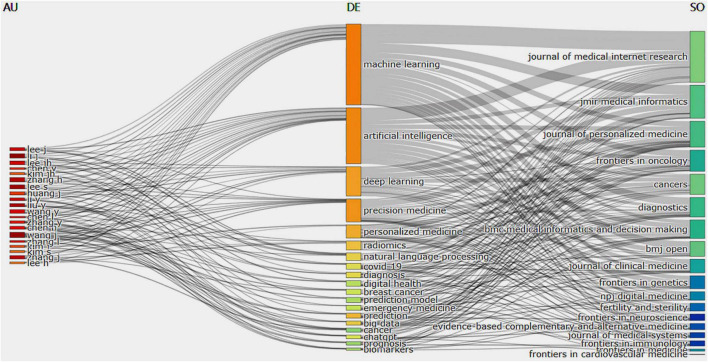
A three-field plot connecting authors (AU), keywords (DE), and sources (SO).

A three-field plot in Figure 9 presents the co-occurrence relationships among authors, keywords, and publication sources. The size of each node signified its frequency or importance, and the connections between nodes represented co-occurrence relationships, with the thickness of the lines indicating how often or strongly these co-occurred. The three categories, listed from left to right, are authors, keywords, and associated journals. The most notable co-occurrence was between “machine learning” and the Journal of Medical Internet Research. The keyword “Artificial intelligence” is mainly associated with the Journal of Medical Internet Research, whereas “deep learning” is primarily linked to JMIR Medical Informatics. Prominent authors demonstrate a robust co-occurrence with the keyword “machine learning.” It is noteworthy that certain authors co-occurred with specific keywords significantly: Wang J and Li J with “machine learning,” Liu Y with “artificial intelligence,” and Lee S with “deep learning.” Based on these observations, we highlight key authors and teams in this area of research, revealing significant co-occurrence with the keyword “machine learning” and suggesting potential future research directions. Keywords co-occurred heavily with certain authors: Wang J and Li J with “machine learning,” Liu Y with “artificial intelligence,” and Lee S with “deep learning.” Research directions can be derived from these observations, which highlight key authors and research teams in the research domain.

**FIGURE 10:**
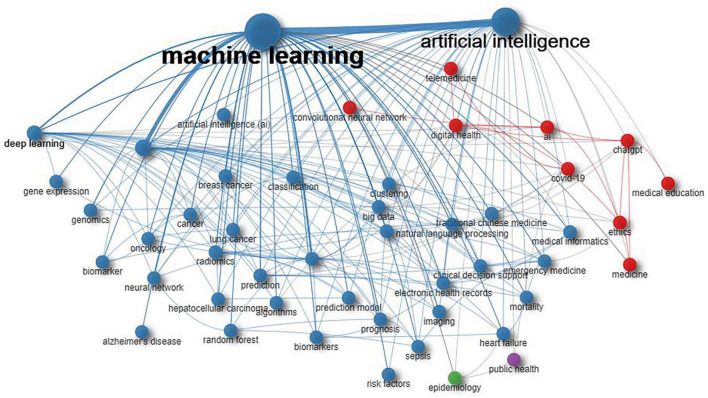
Co-occurrences of keywords were used to cluster research topics.

By analyzing keyword co-occurrences, we were able to identify popular topics and assist scholars in gaining a deeper understanding of current scientific concerns. Developing a co-occurrence network from authors’ keywords helps to pinpoint semantic similarities between terms and reveals the knowledge structures in the relevant field (20). The parameters mentioned in “2 Materials and methods” section served to explain the network illustrated in Figure 10. The authors’ keywords (nodes) are depicted in Figure 10, categorized into four communities (clusters or subdomains). The grouping of terms within a cluster reflects their contextual similarity and relationship. Within the network, edges signify terms that co-occur in the same document. In the red cluster, there are nine terms with primary keywords including “digital health,” “COVID-19,” and “AI.” This cluster explores AI applications in digital health, COVID-19, and ChatGPT. The blue cluster, comprising 37 nodes, prominently features keywords like ML, AI, precision medicine, and deep learning, focusing on AI applications in precision medicine. The green cluster explores AI applications in epidemiology, whereas the fourth cluster focuses on AI applications in public health.

## 4 Discussion

In this research, we performed a bibliometric analysis of relevant literature concerning the application of AI in medicine spanning from 2019 to 2023. Trends over the specified time frame were discerned by tracking publications per year. Articles with high citation counts, identified as those in the top percentile, were recorded because they frequently signify significant scientific advancements. The productivity of countries was assessed based on the volume of AI in medicine publications across the time frame. Keyword analysis was used to identify current research themes and topics of interest. Tracking these bibliometric parameters over time allowed for the visualization of the growth of global AI in medicine research. As represented in Figure 2, there were only 144 articles released in 2019, which suggests a nascent stage of understanding among researchers. The volume of research papers has consistently increased, averaging an annual growth rate of 28.4%. This trend indicates a mounting scholarly interest in the study of AI in medicine, solidifying its status as a popular and enduring research topic. The timeframe includes the emergence of key applications of AI in medicine, such as digital health, precision medicine, and the response to the COVID-19 pandemic, which have significantly impacted healthcare delivery and research priorities.

Based on the review of nations, the United States played the most crucial role in NP and NC, indicating its leadership role in AI. United States President Donald Trump issued the executive order “Maintaining American Leadership in Artificial Intelligence” on 11 February 2019, directing federal agencies to accelerate AI research and development (25). With USD 10,202 spent on healthcare per person, the United States outspends any other nation (26), which may help to explain why there are more publications there. Given the United States has significantly impacted on this academic field and because the majority of partnerships on AI in medicine, there is a clear need for increased international collaboration with the United States. Because of the country’s degree of economic development and the support of national policy, the majority of the pertinent publications were published by China. While China’s AC was relatively low. These findings indicate a disparity in China’s output quantity and quality. Researchers should, therefore, direct their attention to this matter. This challenge requires enhanced collaboration with different nations, including the United States, England, and Germany, while actively tracking scientific progress and performing thorough research.

Another implication involves the factors influencing research output in medical AI. Empirical data indicate that influential authors, identified by total and per-paper citations, are typically those who either lead a field with sustained productivity or develop widely applicable methodologies (27). The productivity of junior and inexperienced authors is also significantly improved by senior and productive authors (28).

At the institutional level, Harvard Medical School has been recognized as the leading institution due to its high volume of published articles in this field, signifying its paramount importance and activity in this domain. In China, Shanghai Jiao Tong University, Sichuan University, and South China University have achieved notable prominence, despite having limited ties to the United States. Research institutions worldwide must collaborate extensively to advance global research in this field. This collaboration will help understand the similarities, differences, and correlations with medicine among various racial groups.

Based on the journal analysis, research on AI in medicine tends to be prominent in large-scale journals related to comprehensive medical topics and internet-related issues. An NC metric measures the influence of a journal, whereas an H metric measures both publication volume and citations (29). The Journal of Medical achieved the highest H-index. In this journal, discussions focus on novel systems techniques and methods for managing hospitals and clinics, pathology, radiology, pharmaceutical delivery, medical records, and patient support systems. It delivers insightful essays, articles, and studies on a variety of medical systems, from large-scale hospital programs to inventive small-scale services. NPJ Digital Medicine, a peer-reviewed open-access journal by Nature Partner Journals (Nature Publishing Group), focuses on digital medicine research. It covers medical information technology, telemedicine, medical sensor technology, health data analysis, and AI applications in healthcare. The journal seeks to promote innovation in digital medicine and offers a platform for researchers to share original research and review articles to advance medical science. Bradford’s law identifies the Journal of Medical, Cancers, BMJ Open, JMIR Medical Informatics, and Journal of Personalized Medicine as key resources for future research, providing valuable references for upcoming scholars. Nevertheless, this legislation focuses solely on the number of publications in pertinent categories, disregarding the quality and impact of the research (30). As a result, it is important to merge research results from various publications to deepen insights into AI applications in medicine.

Keywords provide a concise summary of an article’s core ideas and are key indicators of research directions and hotspots in a certain field. A shift in keywords as time progresses often indicates the evolution of research hot spots and can be used to guide future research. Figure 8 demonstrates a gradual transition in keywords from AI-based classification and its application in cancer diagnosis and disease prediction to outcomes and risk. “Classification” emerged as the most frequently used keyword. Sidey-Gibbons and Sidey-Gibbons (31) developed three ML models to classify breast cancer. The algorithms included Regularized General Linear Model regression (GLM), SVM with radial basis function kernels, and single-layer Artificial Neural Networks. The authors trained these algorithms on the evaluation sample data and then used them to predict diagnostic outcomes in the validation data set. They compared the model predictions on validation datasets with actual diagnostic decisions to calculate accuracy, sensitivity, and specificity. The integration of advanced technologies such as AI in cancer detection and prediction has led to significant advances and diversified applications. In dermatology, combining conventional neural networks with transfer learning techniques has effectively classified skin lesions, providing a valuable tool for early skin cancer detection (32). AI applications in abdominal cancers now include cancer detection, diagnosis, classification, genomics, genetic alteration detection, tumor micro-environment analysis, predictive biomarker identification, and follow-up (33). By analyzing histopathology images, ML can accurately predict patient prognosis in the context of lung cancer, contributing to precision oncology (34). Furthermore, the application of AI has achieved significant success in medical image-based cancer diagnosis, demonstrating favorable results in image classification, reconstruction, detection, segmentation, registration, and synthesis (35). Since 2022, there has been a significant rise in the use of keywords like “Outcomes” and “Risk,” indicating of the integration of AI in medicine. This integration aims to enhance the dialogue between healthcare providers and patients regarding potential health risks and probable outcomes.

Identifying keywords and their evolution in various articles through co-occurrence analysis aids in exploring the focal points within the research field (36). The red cluster explores AI applications in digital health and COVID-19. Digital health refers to the utilization of digital technologies to enhance human health (37). Evidence suggests that AI can utilize electrocardiograph signals to predict atrial fibrillation and select patients for intervention (38). Consequently, this approach allows for targeted therapies addressing actual necessary conditions and diseases. Explainable AI can predict Alzheimer’s disease in patients with mild impairments by using interpretable machine-learning algorithms to elucidate complex patterns for individual patient predictions (39). The focus of digital health has shifted beyond the mere diagnosis and treatment of diseases to encompass early prevention, precision intervention, and health management with the citizen at the center. Innovative AI technology can advance intelligent telemedicine and support the creation of a comprehensive digital health platform, potentially guiding future research.

The COVID-19 pandemic has profoundly impacted the world in unprecedented ways. Effectively managing the pandemic necessitates accurate and timely information regarding the spread of the SARS-CoV-2 virus, the efficacy of mitigation interventions, and its impact on diverse populations. Numerous previous studies have explored the use of AI in combating COVID-19. Hussain et al. (40) found AI showed to be a powerful tool for predicting, detecting, and reducing infectious disease outbreaks in the course of the COVID-19 pandemic. Additionally, Pham et al. (41) and Nguyen et al. (42) discussed the use of AI in vaccine and drug development. During the COVID-19 pandemic, AI has been widely employed to facilitate various tasks. Robots have been utilized for the efficient distribution of essential food items and for disinfecting areas using ultraviolet rays, thereby reducing human exposure to the virus (43). In hospitals, robots have taken over tasks traditionally performed by healthcare workers, thereby alleviating the burden on medical staff. Furthermore, hospitals have been equipped with 5G-powered temperature-detecting devices, and wearable accessories such as wristbands have been utilized for monitoring heart rates and detecting oxygen levels (44). Additionally, robots have assisted patients during quarantine, enhancing the overall experience. Robots have been utilized for patient health monitoring, conducting scans, and sharing data with researchers via cloud services (44). Their immunity to disease and ease of disinfection makes them effective in laboratory testing and clinical trials. Moreover, robots have served as intermediaries between patients and doctors, thereby minimizing the risk of virus exposure to healthcare professionals (45).

ChatGPT, created by OpenAI, is a large language model capable of analyzing and generating text in a way that resembles human intelligence (46). Merely a few months following its release, ChatGPT has already made a significant impact in the field of medical science, embracing scientific research, medical education, medical writing, and diagnostic decision-making (47, 48). ChatGPT is capable of generating scientific articles using suitable vocabulary and varying tones from informal to highly technical (49). By providing accurate differential diagnoses and insights for cancer screening, ChatGPT aids physicians in clinical decision-making (50–52).

In the past decade, AI research has significantly improved the forecasting, identification, diagnosis, categorization, treatment, and survival forecasting of diseases (53, 54), fostering medical innovation and promoting a sustainable approach to precision medicine. Biomarker tests or tools indicate normal biological processes, pathogenic processes, or predictive biomarkers are measured once to forecast future events, while monitoring, response, and safety biomarkers are measured over time (55).

AI applications have exponentially grown across various fields, with the earliest recorded automated pattern recognition appearing in a 1960 report in The Lancet (56). Precision medicine approaches are already being implemented in the context of cancer, encompassing both diagnosis and treatment. In the realm of cancer diagnosis, current literature (57–59) showcases numerous studies delving into AI’s potential, comparing its results to manual detection by pathologists. AI exhibits superior accuracy compared to human pathologists in diagnosing certain cancer types (60–62). The precision medicine approach tailor’s cancer treatment plans by considering tumor-associated and inherited genetic variations, environmental exposures, lifestyle, general health, and medical history. AI is revolutionizing drug discovery, target identification and clinical trials. Traditional methods used in drug discovery are often expensive and time-consuming, and they may not consistently offer accurate forecasts of a drug’s efficacy and safety. The use of AI algorithms, particularly ML models, has significantly advanced drug discovery through better predictive analytics and target identification. By analyzing vast amounts of data, these algorithms hasten the early stages of drug development by detecting patterns and predicting potential drug candidates (63). AI models, such as those in ML and deep learning, draw on data from genomics, proteomics, chemical structures, and clinical trials to pinpoint drug candidates, evaluate safety, and estimate effectiveness using historical data. By forecasting interactions, toxicity, and pharmacokinetics of compounds, AI allows researchers to prioritize drug candidates for further development (64). Moreover, AI simplifies the process of identifying targets by examining biological data and understanding disease mechanisms. AI-powered strategies, such as network analysis and building knowledge graphs, bring together diverse data sources to highlight promising therapeutic targets (65). AI also integrates multi-omics data effectively, offering a broad understanding of disease pathways and improving target identification accuracy (66). Furthermore, neural networks and other deep learning models rank drug targets by evaluating complex connections between molecular characteristics and disease pathways, aiding in therapeutic intervention (67). To find actionable therapeutic targets in amyotrophic lateral sclerosis (ALS), Pun et al. (68) integrated various bioinformatic and deep learning models, which were trained on disease-specific multitopic and text data to prioritize drug-gable genes, revealing 18 potential targets for ALS treatment. Additionally, West et al. (69) created a deep learning method with an innovative modular structure to pinpoint human genes connected to multiple age-related diseases by studying patterns derived from gene or protein attributes such as Gene Ontology terms, protein–protein interactions, and biological pathways. Through automating the identification of suitable participants, AI also has the potential to improve patient recruitment and eligibility in clinical trials. AI algorithms process electronic health records, medical literature, and additional healthcare data to pinpoint potential candidates. Automated prescreening tools help to minimize manual tasks and increase efficiency in recognizing eligible participants (70). Predictive analytics improve recruitment by predicting enrollment rates, enabling trial sponsors to allocate resources efficiently using patient demographics and historical data (71). AI is also altering clinical trial approaches with the help of real-world evidence (RWE) and adaptive trial designs. AI algorithms examine data from various real-world sources to offer a more comprehensive insight into patient demographics, disease development, and treatment results (72). AI supports the division of patients into categories and the recognition of subgroups, targeting distinct patient profiles for adaptive trials, and predictive analytics help in anticipating recruitment rates and treatment responses (73). By adapting trial designs in real-time, protocols are optimized, leading to greater trial efficiency. In general, AI’s adaptive trial designs increase flexibility, efficiency, and success.

Machine learning, a branch of AI, enables computer algorithms to learn from data without being explicitly programmed to carry out tasks (74). As depicted in Figure 10, deep learning has the most significant impact on precision medicine. Deep learning represents the most advanced form of ML presently available and has emerged since 2010 as an AI technique facilitating the analysis of medical images and genomic studies (75, 76). Deep learning has recently used genomic data to identify two unique glioma subtypes, providing insights into their molecular mechanisms (77). Through deep learning, potential candidates can be efficiently screened, therefore reducing drug discovery costs (78). Without explicit instructions, the system discerned patterns from the data and autonomously learned what to seek and report.

Based on the summary of the Public Health and Epidemiology Informatics section in the 2017 IMIA Yearbook (79), precision public/global health and digital epidemiology are still used in 2018 (80, 81). It entails providing the appropriate intervention to the suitable population at the optimal time (80). The latter phrase pertains to employing digital data, especially data not deliberately gathered, to answer epidemiological questions (81). The significant potential of Big Data in epidemiology was showcased by Deiner et al.’s (82) innovative study, which demonstrated that monitoring social media for disease symptom queries can lead to early detection of epidemics. Pattern recognition and data analytics were employed to detect, identify, and categorize patterns of disease occurrence associated with conjunctivitis. Conversely, wearable technologies will enable the monitoring and collection of individual medical information and the refinement of the care process. The fusion of AI with virtual reality and augmented reality (83), will enable the creation of both virtual medical services that citizens can access easily and directly, as well as increasingly effective and safe applications for robotic surgery.

It is noteworthy that the keyword “ethics” is depicted in Figure 10, indicating a growing focus on AI ethics in medicine. Regulatory laws and guidelines for medical AI are frequently formulated without engaging in dialogue among community members, clinicians, developers, and ethicists. This lack of collaboration may result in regulations that do not align with the experiences of community members as users of medical AI. Ethical concerns highlighted by policymakers and scholars may not match those of patients, providers, and developers, leading to a disconnect that makes ethical decision-making tools ineffective for AI users. The ethical issues identified by policymakers and scholars may not correspond with those of patients, providers, and developers, creating a disconnect that makes ethical decision-making tools ineffective for AI users (84). Analyzing empirical studies on the ethics of medical AI assists educators, researchers, and ethicists in understanding and addressing perceived ethical concerns (85). Understanding the ethical awareness of patients, families, and healthcare providers regarding AI in healthcare is essential for informing the progression and research of medical AI. Identifying stakeholders perceived ethical risks of medical AI allows for the development of practice protocols, organizational norms, and legal requirements to promote AI interventions guided by ethical considerations. Although AI has significantly benefited the healthcare system and advanced medicine, unethical use of this technology can endanger both patients and physicians. Establishing ethical standards for all stakeholders in healthcare and related fields is essential. Establishing global and national protocols to regularly review and validate AI products in clinical and practical settings is essential.

Nonetheless, this study still has certain limitations. Firstly, this study only searched the WoSCC database, which is considered one of the most widely used large multidisciplinary abstract databases globally, but it may still have incomplete coverage and the search strategy is not perfect. Secondly, only English-language articles were included. But this limitation is unlikely to affect the study’s stability significantly, as the WoSCC database predominantly features articles in English. Finally, there is a lag in the citation numbers for articles, which means that more recently published high-quality articles may have been under explored. Future studies are recommended to be updated accordingly and the words like these: “Computer Heuristics,” “Expert Systems,” “Fuzzy Logic,” “Knowledge Bases,” “Natural Language Processing,” and “Neural Networks, Computer” are related to the search strategy.

## 5 Conclusion

The study analyzed papers from WoSCC published between 2019 and 2023 on the integration of AI and medicine. Our research shows a significant rise in yearly publications, suggesting growing interest in this subject. A bibliometric analysis shows that the United States leads the world both in the volume of publications and their central role, indicating its paramount importance and activity in this domain. Universities are the primary research institutions in this field. So, there remains a requirement for more effective cross-regional and international cooperation to further drive progress. Recent keyword clustering identifies “digital health,” “COVID-19,” “precision medicine,” and “epidemiology and public health” as emerging research frontiers. It is foreseeable that AI will increasingly play a crucial role in digital health and public health, and has a significantly improvement for the forecasting, identification, diagnosis, categorization, treatment, and survival forecasting of diseases to promote a sustainable approach for precision medicine. This bibliometric study aids researchers in identifying the present state and developing trends in medical AI and is beneficial for optimizing medical resource use and enhancing patients’ quality of life.

## Data Availability

The raw data supporting the conclusions of this article will be made available by the authors, without undue reservation.
